# Assessment of Intraspecific Variability in the Forest Dormouse (*Dryomys nitedula*) and Woolly Dormouse (*Dryomys laniger*) from Türkiye and Adjacent Regions Based on Mitochondrial DNA

**DOI:** 10.3390/life15040660

**Published:** 2025-04-17

**Authors:** Ercüment Çolak, Georgi Markov, Engin Selvi, Teoman Kankılıç, Perinçek Seçkinozan Şeker, Maria A. Kocheva, Milena K. Gospodinova, Reyhan Çolak, Hristo Dimitrov, Nuri Yiğit

**Affiliations:** 1 Department of Biology, Faculty of Science, Ankara University, Ankara 06100, Türkiye; colak@science.ankara.edu.tr (E.Ç.); rcolak@science.ankara.edu.tr (R.Ç.); nygt@science.ankara.edu.tr (N.Y.); 2 Institute of Biodiversity and Ecosystem Research, Bulgarian Academy of Sciences, 1113 Sofia, Bulgaria; georgimar@gmail.com (G.M.); mariakocheva@gmail.com (M.A.K.); milenagos@abv.bg (M.K.G.); 3 Department of Biotechnology, Faculty of Science and Letters, Ömer Halisdemir University, Niğde 51000, Türkiye; teomankankilic@gmail.com; 4 Department of Forestry, Artvin Vocational School, Artvin Çoruh University, Artvin 08100, Türkiye; 5 Department of Zoology, Faculty of Biology, Plovdiv University, 4000 Plovdiv, Bulgaria; hr_dim@abv.bg

**Keywords:** *Dryomys nitedula*, *Dryomys laniger*, intraspecific variation, cytochrome *b*, phylogeography, phylogeny, Türkiye

## Abstract

This study aimed to reveal intraspecific variations in two *Dryomys* species distributed in Türkiye, based on mitochondrial DNA cytochrome *b* gene sequences, and to discuss the factors driving these variations in the context of phylogeography and genetic species concepts. As a result of Maximum Likelihood, Bayesian Inference, and Network analyses, which included haplogroups or lineages from Italy, Russia, the Caucasus, and Iran identified in previous studies, along with Turkish haplotypes, three major clades (MC1, MC2, and MC3) were identified within *Dryomys nitedula*. These clades began to diverge evolutionarily in the middle of the Late Miocene (8.82 million years ago) and exhibit significant genetic differences from one another. The Turkish haplotypes were divided into five distinct lineages (N1–N5), each within five subclades (SC1–SC5), which were nested within these MCs. These lineages, their geographical distributions, and the subspecies defined in previous studies that correspond to these lineages are as follows: N1 from the Thrace region (*Dryomys nitedula wingei*), N2 from the Black Sea region (potentially a new subspecies), N3 from western and central Anatolia (*Dryomys nitedula phrygius*), N4 from northeastern Anatolia (*Dryomys nitedula tichomirowi*), and N5 from eastern Anatolia (*Dryomys nitedula pictus*). The N2 lineage, distributed in areas close to the coastal side of the Eastern Black Sea region and with a range close to both N3 (*D. n. phrygius*) and N4 (*D. n. tichomirowi*), exhibited high genetic differentiation from these two lineages and was a candidate to be treated as a new subspecies of *Dryomys nitedula* in Türkiye. The N5 lineage, which includes haplotypes from the distribution areas of the populations initially classified as *Dryomys pictus* and later as *Dryomys nitedula pictus* in previous studies, was found to be more closely related to *Dryomys nitedula kurdistanicus* from the Zagros Mountains than to *D. n. pictus* from the central regions of Iran. Combining the results of this study with previous research, it is clear that the *D. nitedula* lineages in Türkiye, along with haplogroups or subspecies in neighboring regions diverged between the middle Late Miocene and Middle Pleistocene. This divergence is believed to have been driven by climatic cycles and geomorphological processes that shaped the topography of their distribution range. The high genetic diversity observed in the lineages of Anatolia suggests that the region may have served as a glacial refuge for *D. nitedula*. Similarly to the processes and factors shaping the evolution of *D. nitedula*, *Dryomys laniger* was found to have diverged into two lineages, western (L1) and eastern (L2 or *Dryomys anatolicus*), within its distribution range during the Late Pliocene (2.94 Mya). To make a more accurate taxonomic assessment of *D. laniger*, a larger number of samples is needed, and the distribution limits should be more clearly defined.

## 1. Introduction

*Dryomys nitedula*, known as the forest dormouse, and *Dryomys laniger*, also called the woolly dormouse, are the two extant species of the genus *Dryomys* in Türkiye [1,2]. *Dryomys nitedula* has a wide distribution across Europe, the Caucasus, the Middle East, and Central Asia [1,2,3]. This is an arboreal species and typically inhabits forested areas, such as the Black Sea region, as well as gardens and fragmented forests in Türkiye [1]. The other representative of this genus, *D. laniger* known as woolly dormouse, is endemic to Türkiye. It inhabits areas at elevations ranging from approximately 1400 to 3000 m above sea level, with its geographical distribution extending from the rocky habitats in the Taurus Mountains in southern Türkiye to eastern Anatolia [1,2,4,5].

*Dryomys nitedula* exhibits significant intraspecific diversity across its entire distribution range. In addition to the results from past studies using classical methods such as morphology, karyology, and allozyme analysis, recent studies using molecular markers have also contributed to revealing intraspecific variations of *D. nitedula* to some extent across its global distribution [1,6,7,8,9,10,11,12,13,14,15]. The populations of this species in the Caucasus were morphologically evaluated and classified into four subspecies based on variations in fur color: *Dryomys nitedula daghestanicus*, *Dryomys nitedula caucasicus*, *Dryomys nitedula ognevi* and *Dryomys nitedula tichomirowi* [16]. In another study on the mitochondrial phylogeography and taxonomy of *D. nitedula* populations in the Caucasus, the populations in the Western Caucasus were described as a new subspecies, *Dryomys nitedula heptneri* [17]. A comprehensive study of mitochondrial DNA cytochrome *b* (*CYTB*) variation in *D. nitedula* populations across Russia and the Caucasus identified three distinct haplogroups, each showing high levels of genetic divergence: Eastern European (EE), Western Caucasus (WC), and Central Caucasus (CC) [18]. Also, the study suggested that they were isolated for an extended period in Eastern Europe and the Caucasus during the Pleistocene. Based on the significant genetic differences between the three distinct haplogroups identified, the study proposed that the Caucasian populations of the forest dormouse may merit distinct species status. The study also noted that the geographical distribution of the identified haplogroups corresponded to the ranges of *Dryomys nitedula nitedula* (the nominotypical subspecies) and *D. n. ognevi* [18]. A recent study on mitochondrial and nuclear DNA variations of *D. nitedula* populations in Iran and its surroundings, an area close to Russia and the Caucasus, revealed at least six geographically distinct lineages corresponding to the previously described subspecies based on the morphology and morphometry (*Dryomys nitedula bilkjewiczi* from Kopet Dagh in Iran, *Dryomys nitedula kurdistanicus* from Zagros Mountains in Iran, *D. n. tichomirowi* from Lesser Caucasus in Iran and Georgia, *D. n. ognevi* from Central and Eastern Caucasus and Alborz in Russia and Iran, *D. n. heptneri* from Western Great Caucasus in Russia, and *D. n. pictus* in Central Iran) in this area. Additionally, the study found that *D. n. pictus* lineage was genetically quite distinct from other subspecies, providing strong evidence that *D. n. pictus* may represent a separate species [19]. In addition to the intraspecific variations of the forest dormouse observed in Russia, the Caucasus, and Iran, lineages corresponding to two subspecies (*Dryomys nitedula intermedius* and *Dryomys nitedula aspromontis*) were identified in Italy [20]. These lineages were genetically quite distinct, and it was suggested that the populations in southern Italy may represent a previously unknown new species [20].

Studies on *Dryomys nitedula* populations in Türkiye, a transitional region between Europe and the Near East, revealed the presence of four distinct subspecies in the Türkiye: *Dryomys nitedula wingei* in Thrace, *Dryomys nitedula phrygius* (with the type locality in Uşak–Muratdağı) in central and western Anatolia, *D. n. pictus* in eastern Anatolia, and *D. n. tichomirowi* in northeastern Anatolia [1,4,21]. Populations of *D. nitedula* in the Hakkari–Cilo Mountains and its surroundings in the southeastern part of Türkiye have been recognized as a distinct third species, *Dryomys pictus*, within the genus *Dryomys* [22]. However, subsequent studies on these populations suggested that *D. pictus*, previously considered a separate species, is a subspecies of *D. nitedula* (*Dryomys nitedula pictus*) in fact [1,2]. Recent molecular studies aimed at determining intraspecific variations have revealed the existence of two to four geographically distinct genetic groups within *D. nitedula* in Türkiye. Two studies based on allozyme variations produced conflicting results. In one study, the Thrace and Anatolian populations of *D. nitedula* were grouped together [8], while in the other, populations from Thrace, central Anatolia, and northeastern Anatolia were separated [4]. A study based on mitochondrial DNA *ND1* gene sequences, along with a subsequent study combining Beta–fibrinogen intron 7 (*Fib7*) and 12S ribosomal RNA (*12S rRNA*) sequences, produced similar results. Both studies revealed that *D. nitedula* in Türkiye is divided into four distinct lineages: Thrace, Anatolia, northeastern Anatolia, and Şavşat [6,15]. A recent study based on whole mitochondrial genome sequencing of *D. nitedula* in Türkiye revealed that the species is divided into at least two lineages. The study suggested that increasing the sample size and using additional molecular markers would be necessary to fully elucidate intraspecific relationships [23].

The identification of approximately 20 subspecies within its distribution range, along with the presence of two main genetic groups in Italy, three in Russia and the Caucasus, four in Türkiye, and at least six in Iran and adjacent areas, clearly indicates the high intraspecific genetic diversity of *Dryomys nitedula* [1,2,4,6,15,17,18,19,20,24,25]. All available molecular studies have suggested that populations exhibiting high levels of genetic diversity within this species could represent a separate species [6,15,17,18,19,20]. However, these studies have been inconclusive, as they employed different molecular markers and lacked comprehensive sampling across the species’ entire global distribution. Therefore, further studies are needed to gain a clearer understanding of the species’ intraspecific variations on a global scale. As it has been suggested by previous studies, this can be achieved by selecting methods that align with those used in past studies, allowing for the regional results to be evaluated in conjunction with global data [6,15,18,19]. In addition to previous studies on European and the Near East populations using mitochondrial DNA *CYTB* sequences as molecular markers [18,19,20], a study on the populations of this species living in Türkiye, which is within the distribution area of the species and acts as a biogeographic bridge between Europe and the Near East, using the same molecular marker may provide unifying results to better understand the current uncertainties arising from high intraspecific variability.

Few studies on *Dryomys laniger*, a species endemic to Türkiye, have explored its ecological and biological characteristics, including habitat, karyology, and morphology [3,4,10]. Two recent studies have provided mitochondrial and nuclear DNA sequences, though based on a limited number of samples [6,15]. Other studies found that *D. laniger* is divided into two distinct lineages within its distribution range, western and eastern, based on variations in mitochondrial and nuclear gene sequences [23,26]. Additionally, samples collected from Kahramanmaraş in southeastern Türkiye and Tunceli in eastern Anatolia were identified as a new species, *Dryomys anatolicus*, based on differences in mitochondrial and nuclear gene sequences [26]. However, there is a significant distance between the two locations, Kahramanmaraş and Tunceli, where *D. anatolicus*, introduced as a new species, was described. DNA-based studies on samples collected from the area between these two locations could help validate the existence of the new species, *D. anatolicus*.

Türkiye is a unique region with a rich biological diversity, shaped by some factors such as its geographical location, climatic variety, topographic features, and being located in an active tectonic area [27]. Additionally, Türkiye is considered an important glacial refuge that played a significant role in the formation and shaping of the present-day biodiversity of the European continent [28,29,30]. In this context, characterizing the genetic structures of species in Türkiye and comparing them with their European counterparts by revealing their genetic diversity levels is crucial for providing evidence to test this consideration. Mitochondrial DNA is a valuable marker for microevolutionary phylogenetic studies, owing to its high copy number, lack of genetic recombination, maternal inheritance, and rapid evolutionary rate. These characteristics make mitochondrial genes widely used to explain changes in population genetic structure across time and space [31]. The sequence variability of the cytochrome *b* (*CYTB*) gene makes it particularly well suited for comparing species within the same genus or family [32]. The *CYTB* aligns more closely with traditional mammalian phylogeny and exhibits greater base diversity within a shorter sequence. If a locus is to be used as a standard for phylogeny and species identification in mammals, the *CYTB* gene is the preferred choice [33]. The primary aim of this study is to explore intraspecific variation in *Dryomys* species in Türkiye through extensive sampling and mitochondrial DNA *CYTB* gene sequence analysis. This will allow for the identification of the main lineages or genetic groups (haplogroups) within two species (*Dryomys nitedula* and *Dryomys laniger*), and an examination of the factors that shape these lineages from both a phylogenetic and phylogeographic perspective. Based on the identified lineages, the validity of recognized subspecies or species will be assessed, contributing to the clarification of the species’ taxonomic status. Additionally, by analyzing all mitochondrial DNA *CYTB* lineages of *D. nitedula* from previous studies alongside the lineages found in Türkiye, an important land bridge between Europe and Asia, the phylogeographic cohesion of *D. nitedula* will be further established.

## 2. Materials and Methods

### 2.1. Sampling, DNA Extraction, and Amplification

**Figure 1:**
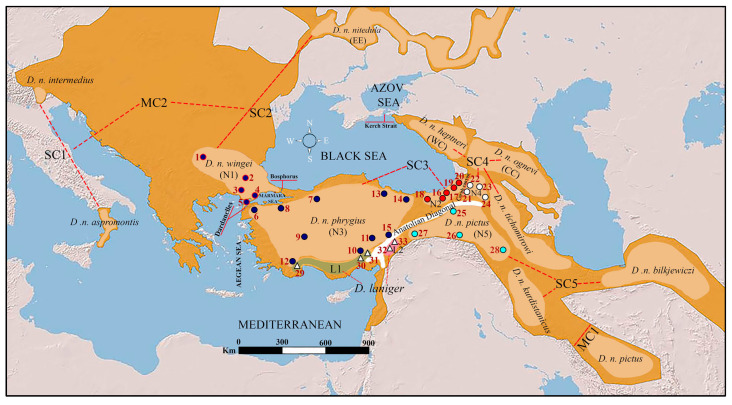
The map shows the collection sites of *Dryomys nitedula* and *Dryomys laniger* samples in Türkiye and Bulgaria, along with sampling sites from previous studies in Italy, Russia, Caucasus, and Iran. The numbers, circles and triangles on the map correspond to the localities listed in Table A1 and Table A2. Red dotted lines represent the connections between major clades and their subclades. Please refer to the main text for a more detailed explanation.

A total of 88 *Dryomys nitedula* samples (1 from Bulgaria and 87 from 28 different localities across Türkiye) and 22 *Dryomys laniger* samples from 5 different localities in Türkiye were used in this study (Figure 1 and Table A1). All samples were collected under a permit by the Ministry of Agriculture and Forestry, Republic of Türkiye, with the date and number as follows: 02/07/2018–72784983–488.04–150036. Experimental studies were conducted under the decision of the Ethics Committee of Ankara University (Ankara, Türkiye) with the date and number as follows: 06/06/2018–2018–12–80. *Dryomys nitedula* is classified as Least Concern (LC) on the IUCN Red List, while *Dryomys laniger* is classified as Data Deficient (DD). While *Dryomys nitedula* is an arboreal species, *Dryomys laniger* primarily inhabits rocky mountainous areas. Since both species hibernate, field studies were primarily conducted during their active period, from early spring to late summer. During field studies in Thrace, *D. nitedula* samples were collected from various habitats at elevations near sea level (20–150 m), including agricultural areas with fruit trees along the edges, mixed dense vegetation near streams, and fruit and vegetable gardens. Samples collected from western and central Anatolia were obtained from the entrances of mixed deciduous forests with various forest fruits along the edges, at altitudes ranging from approximately 900 to 1800 m above sea level. Samples collected from the Eastern Black Sea region were obtained from mixed forests on the northern slopes overlooking the Black Sea coast, at altitudes ranging from 500 to 1500 m. Samples collected from northeastern Anatolia were obtained from forests extending south of the Çoruh Basin to Posof (Ardahan) and Kars, with altitudes ranging from 700 to 2000 m. Samples collected from the eastern Anatolia region were obtained from areas located east of the mountain range known as the Anatolian Diagonal, at altitudes ranging from 1000 to 2000 m. *Dryomys laniger* samples were collected from rocky areas with short, dwarf shrubs and occasional trees, at altitudes ranging from approximately 1500 to 2000 m (Figure 1 and Table A1).

The CTAB isolation protocol was used to obtain total genomic DNA from various tissues of the samples [34]. Polymerase chain reactions including L14724a and H15915R [35] primer pairs were performed for the amplification of cytochrome *b* gene region. The total volume of the reaction mix was 37.5 µL containing 21.9 µL sterile water, 3.75 µL buffer (750 mM Tris–HCl pH 8.8 at 25 C, 200 mM (NH_4_)_2_SO_4_, 0.1% Tween 20, Fermentas, Waltham, MA, USA), 6 µL dNTP mix (A, C, G, T 200 mM), 3 µL MgCl_2_ (25 mM), 0.45 µL forward and reverse primers (20 pmol, Fermentas, Waltham, MA, USA), 0.45 µL Taq polymerase (500 U, Fermentas, Waltham, MA, USA), and 1.5 µL DNA (at least 500 ng/µL for each sample) samples. Amplification reactions were carried out in BIO-RAD Thermal Cycler (Hercules, CA, USA) and started with an initial denaturation step at 94 °C for 5 min, followed by 30 cycles: denaturation for 1 min at 93 °C, primer annealing for 1 min at 50 °C, extension for 5 min at 72 °C, and final extension for 1 min at 72 °C. After amplification, PCR products were run in 70 V for 90 min in 1 X TAE on 1% agarose gel. Thermo Scientific GeneRuler (Waltham, MA, USA), 100 bp DNA Ladder marker system, was used to detect the estimated size of each DNA sample on agarose. The agarose gels were stained in ethidium bromide (EtBr) solution for 15 min, and later, Major Sciences visualization system (Taoyuan, Taiwan) was employed for imaging of all gels.

### 2.2. Phylogenetic Analyses, Molecular Dating, and Genetic Structure Assessments

Editing, alignment, and consensus sequence generation were performed using BioEdit version 7.0.5.3 [36]. A total of 941 bp of the *CYTB* gene sequences from *Dryomys nitedula* and *Dryomys laniger* were analyzed. For outgroup comparisons, *CYTB* gene sequences from various rodent species were obtained from the National Center for Biotechnology Information (NCBI) (Table A2). Maximum Likelihood (ML) analysis was conducted using MEGA X version 10.1 [37], while Bayesian Inference (BI) was performed using BEAST v1.8.0 [38] for phylogenetic, phylogeographic, and molecular dating analyses. Based on the Bayesian Information Criterion (BIC) and corrected Akaike Information Criterion (AICc), TN93 + G + I was selected as the most appropriate nucleotide substitution model as determined by MEGA X version 10.1 [37]. The robustness of the phylogenetic reconstructions was assessed using a nonparametric bootstrap test with 1000 replicates for ML and Bayesian posterior probability (BPP) for BI.

For molecular dating analysis, three independent MCMC (Markov Chain Monte Carlo) runs of 10,000,000 generations were performed in BEAST v1.8.0 [38], with a sampling frequency every 1000 generations. The effective sample size (ESS) lower bound was checked to ensure it exceeded 200 for three independent runs using Tracer v1.6 (part of the BEAST package). The three separate tree files obtained containing 30,000 trees were combined by LogCombiner v1.8.0 (part of BEAST package). The maximum clade credibility tree with mean heights was generated using TreeAnnotator v1.8.0 (part of the BEAST package). To construct the tree, the initial 10% of sampled trees were discarded as burn-in, and a 50% majority rule consensus tree was created to estimate Bayesian posterior probabilities (BPPs) and divergence times for the tree nodes. Two divergence times were used as calibration points: the *Mus*/*Rattus* divergence at 11.7 ± 0.4 million years [39], and the *Eliomys quercinus*/*Eliomys melanurus* divergence at 7 ± 0.9 million years [40,41]. The Bayesian tree was visualized using FigTree v1.8.0 [42]. Additionally, the evolutionary relationship networks of the populations were constructed using the median-joining method [43] in Network version 10.2.0.0 (Fluxus Technology, Colchester, UK).

DNA polymorphism, number of haplotypes, haplotype diversity, nucleotide diversity, and their standard deviations were calculated using DNAsp v6 [44]. The analysis of molecular variance (AMOVA) was performed using Arlequin version 3.5.1.2 [45] to identify the sources of variation and investigate the genetic structure of subpopulations. Genetic distances between populations were calculated using the Kimura–2-Parameter (K2P) model with 1000 bootstrap replicates in MEGA X version 10.1 [37].

## 3. Results

Based on the variation in the 941-bp *CYTB* sequences from 88 *Dryomys nitedula* and 22 *Dryomys laniger* samples, a total of 47 unique haplotypes were identified for *D. nitedula*, and 8 for *D. laniger*. Detailed information on the haplotypes was provided in Table A2. Multiple sequence alignments revealed 723 invariable sites, 218 variable sites, 206 parsimony-informative sites, and 12 singleton variable sites in *D. nitedula*. In contrast, *Dryomys laniger* had 877 invariable sites, 64 variable sites, 59 parsimony-informative sites, and 5 singleton variable sites. For the *Dryomys nitedula* sequences, the estimated average transition to transversion ratio (R) was 9.22, based on the HKY + G + I nucleotide substitution model. The nucleotide frequencies were A = 29.4%, T/U = 28.6%, C = 27.9%, and G = 14.1%. For *Dryomys laniger* sequences, analyzed using the HKY + G nucleotide substitution model, the R was 7.37, with nucleotide frequencies of A = 29.8%, T/U = 29.9%, C = 26.4%, and G = 13.9%.

### 3.1. Phylogeny

**Figure 2:**
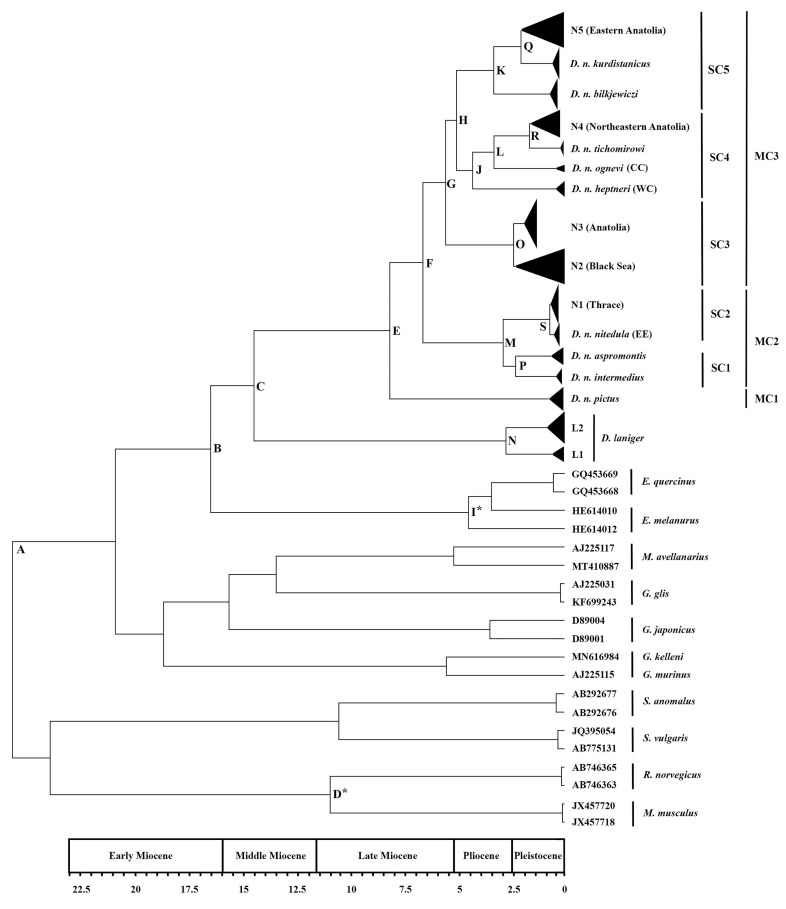
Phylogenetic tree and divergence dating results based on the Bayesian Inference analyses. A–S on the nodes of the phylogenetic tree show the evolutionary divergence times among major clades, subclades, and lineages of *Dryomys*. Triangle sizes are proportional to the sample sizes of the major clades and their subclades. Asterisks (*) on the nodes indicate the calibration points. The ML phylogenetic tree revealed a similar topology (Figure A1). For specific node ages, posterior probability values, 95% highest posterior densities (HPDs) and K–2P distances for the main nodes, see Table 1.

**Figure A1:**
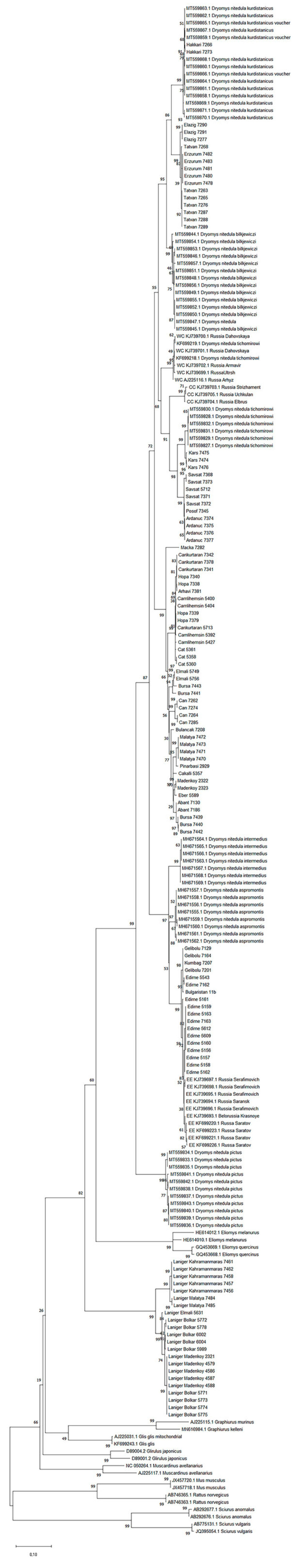
Phylogenetic relationships between *Dryomys* lineages based on ML analysis using *CYTB* data set.

The phylogenetic trees based on Maximum Likelihood (ML) and Bayesian Inference (BI) showed similar topologies, except for the position of *Eliomys* (Figure 2 and Figure A1). In the BI tree, *Eliomys* was a basal group outside the *Dryomys* species, while in the ML tree, it clustered between *Dryomys laniger* and *Dryomys nitedula*. The study identified 96 *D. nitedula CYTB* haplotypes, grouped into three major clades (MC1, MC2, and MC3) and five subclades (SC1–SC5), including haplotypes from Türkiye, Italy, Russia, the Caucasus, and Iran. The phylogenetic tree also revealed five intraspecific lineages in *D. nitedula* (N1–N5) and two in *D. laniger* (L1 and L2) from Türkiye. These major clades, subclades, and lineages corresponded with the geographic distributions of previously described *D. nitedula* subspecies and genetic groups of *D. laniger*.

MC1 included a single lineage from Central Iran: *Dryomys nitedula pictus*. MC2 contained the following lineages: the Italian lineages (*Dryomys nitedula intermedius* from northeastern Italy and *Dryomys nitedula aspromontis* from southern Italy) in SC1, and N1 (*Dryomys nitedula wingei* from Thrace, the European part of Türkiye) along with EE (*Dryomys nitedula nitedula* from Belorussia and Russia) in SC2. MC3 consisted of the following *CYTB* lineages: N2, a potential new taxon from Black Sea region of Türkiye, N3 (*Dryomys nitedula phrygius* from the most of Anatolia) in SC3, WC (*Dryomys nitedula heptneri* from western Caucasus), CC (*Dryomys nitedula ognevi* from Central Caucasus), *Dryomys nitedula tichomirowi* from northwestern Iran and Georgia, N4 (*Dryomys nitedula tichomirowi* from northeastern Anatolia) in SC4, *Dryomys nitedula kurdistanicus* from Zagros Mountains in the western Iran, *Dryomys nitedula bilkjewiczi* from Kopet Dagh in the northeastern Iran, and N5 (previously described as *Dryomys nitedula pictus* from eastern Anatolia) in SC5 (Figure 2).

In Türkiye, five distinct *Dryomys nitedula* lineages were identified. The first lineage, N1, included haplotypes from Thrace and clustered with *D. nitedula* from Eastern Europe and Italy. The second lineage, N2, comprised haplotypes from the coastal side of Eastern Black Sea region. The third lineage, N3, extended across most of Anatolia, from western and central Anatolia to the Eastern Black Sea region. The fourth lineage, N4, consisted of haplotypes from northern east Anatolia, grouping with *D. n. tichomirowi* from northwestern Iran and Georgia, with *D. n. ognevi* and *D. n. heptneri* from the Caucasus as basal groups. The fifth lineage, N5, included haplotypes from east Anatolia, with *D. n. kurdistanicus* and *D. n. bilkjewiczi* as basal groups (Figure 2).

**Figure 3:**
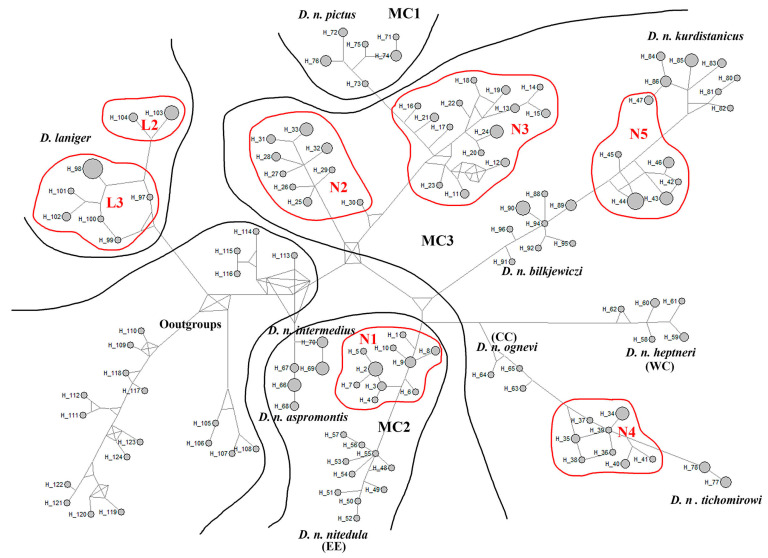
The median-joining haplotype network reconstructed based on partial *CYTB* sequences displays the relationships among major clades, subclades, and lineages of *Dryomys* species. Red-lined haplotypes indicate *Dryomys* lineages in Türkiye. The circles are proportional to the sample size. Explanations of the haplotypes were identified in Table A2.

For *Dryomys laniger*, two distinct lineages were identified: L1, from the West and Central Taurus Mountains, and L2, from the western Southeast Taurus Mountains. The median-joining network (Figure 3) and phylogenetic trees confirmed the presence of these five *D. nitedula* and two *D. laniger* lineages in Türkiye, supporting the observed topologies (Figure 2 and Figure A1).

### 3.2. Divergence Time

Molecular dating using Bayesian Inference estimated that the divergence between *Dryomys nitedula* and *Dryomys laniger* occurred around 15.68 million years ago (Mya) during the Middle Miocene. The initial diversification of *Dryomys nitedula* lineages began approximately 8.82 Mya in the Late Miocene. The first lineage from Türkiye, N1, split from other lineages around 7.15 Mya. The other four *Dryomys nitedula* lineages in Türkiye diverged at the following times: 6 Mya, 5.45 Mya, 4.64 Mya, 3.57 Mya, and 2.56 Mya, spanning from the Miocene–Pliocene border to the start of the Pleistocene. The two *Dryomys laniger* lineages (L1 and L2) began to diverge around 2.89 Mya during the Pliocene–Pleistocene transition, nearly coinciding with the divergence of *D. nitedula* lineages N2 and N3. The divergence between *Dryomys nitedula* and *Dryomys laniger* was moderately supported (BPP = 0.72), and the divergence times for other nodes were strongly supported by high BPP values (Table 1).

**Table 1:** Estimates of the evolutionary divergence times among major clades, subclades, and lineages of *Dryomys nitedula* and *Dryomys laniger* from Türkiye along with the previously determined *D. nitedula* haplotypes from Italy, Russia, Caucasus, and Iran based on BI analysis. The nodes (A–S) in the table were also shown in Figure 2. NA: node age, Myr: millions of years, HPD: high posterior density, BPP: Bayesian posterior probability, K–2P: Kimura–2-Parameter distance, Asterisks (*) indicates calibration points.

Nodes	Description	NA (Myr)	%95 HPD (Myr)	BPP	K2P (%)
A	*Sciuridae* + *Mus* + *Rattus*/*Gliridae*	27.88	22.68–34.24	1	–
B	*Eliomys*/*Dryomys*	17.87	14.1–22.15	1	–
C	*D. laniger*/*D. nitedula*	15.68	12.28–19.49	0.72	20.3
D *	*Mus*/*Rattus*	11.82	11.04–12.58	1	–
E	*D. n. pictus* (MC1)/*D. nitedula* (MC2 + MC3)	8.82	6.79–11.12	1	12.9
F	MC2/MC3	7.15	5.56–8.96	0.99	10.4
G	SC3/SC4	6.00	4.64–7.46	0.99	–
H	SC4/SC5	5.45	4.21–6.79	0.88	–
I *	*E. melanurus*/*E. quercinus*	4.84	3.57–6.14	1	–
J	WC/CC + *D. n. tichomirowi* + N4 (northeastern Anatolia)	4.64	3.55–5.86	0.99	7.19
K	*D. n. bilkjewiczi*/*D. n. kurdistanicus* + N5 (eastern Anatolia)	3.57	2.67–4.63	1	5.47
L	CC/*D. n. tichomirowi* + N4 (northeastern Anatolia)	3.56	2.67–4.59	1	6.94
M	SC1/SC2	3.09	2.07–4.36	1	5.49
N	L1–L2	2.94	2.01–3.96	1	5.57
O	N2 (Black Sea)/N3 (central Anatolia)	2.56	1.89–3.31	1	4.01
P	*D. n. intermedius*/*D. n. aspromontis*	2.46	1.51–3.58	0.61	4.27
Q	*D. n. kurdistanicus* + N5 (eastern Anatolia)	2.12	1.57–2.8	1	4.90
R	*D. n. tichomirowi*/N4 (northeastern Anatolia)	1.77	1.24–2.36	1	4.72
S	EE/N1 (Thrace)	0.74	0.47–1.06	1	1.15

### 3.3. Genetic Diversity

To characterize the genetic structure of both species, estimates of segregating sites, the number of haplotypes and both haplotype and nucleotide diversities (along with their standard deviations) were calculated for all lineages identified in the phylogenetic analyses. The results of these analyses were summarized in Table 2. Among the *Dryomys nitedula* lineages, the highest nucleotide diversity was found in the N3 lineage, while the lowest was observed in the N1 lineage. In total, it was found that the four Anatolian lineages (N2 + N3 + N4 + N5) exhibited approximately 7.5 times greater nucleotide diversity than the Thracian lineage (N1). Additionally, haplotype diversity was high across all *D. nitedula* lineages, whereas the two *D. laniger* lineages (L1 and L2) displayed moderate haplotype diversity and relatively low nucleotide diversity. The AMOVA, conducted at three hierarchical levels, revealed that a large portion of the variation (77.28%) in the mitochondrial DNA *CYTB* gene sequences of *Dryomys nitedula* was due to genetic differences among lineages. Variation among populations within lineages accounted for a smaller proportion (17.81%), while the lowest level of variation was observed within populations (4.92%). The average K–2P distances revealed significant genetic divergence between the nodes, with a 20.3% distance separating *D. nitedula* and *D. laniger*, confirming their classification as two distinct biological species under the genetic species concept. Within *Dryomys nitedula*, the average K–2P distances ranged from 4.01% to 11.19%, while the distance between the two *Dryomys laniger* lineages was 5.57% (Table 1 and Table 3).

## 4. Discussion

This study focused on the intraspecific variations of *Dryomys nitedula*, which is widely distributed across the Palearctic, and *Dryomys laniger*, endemic to Türkiye. It was aimed to uncover the phylogeny and phylogeography of both species by analyzing variations in their mitochondrial DNA *CYTB* sequences. This study combined *CYTB* sequences from previous studies within the distribution range of *D. nitedula* with newly obtained sequences from extensive sampling in Türkiye. This approach enabled a clearer evaluation of the uncertain taxonomic status of previously identified populations, which exhibited high genetic differences, on a global scale. Additionally, the data presented from Türkiye, which serves as a land bridge between Europe and the Near East, were crucial for drawing inferences about the phylogeography of *D. nitedula*. The topologies of the ML and BI phylogenetic trees aligned closely with the geographical distributions of populations, which have been evaluated as subspecies or distinct lineages based on intraspecific variations identified in numerous previous studies. Divergence time estimates based on mitochondrial and nuclear gene sequences, along with the fossil records previously defined [46,47,48,49], yielded consistent results, all suggesting that differentiation within the *Dryomys* genus began during the Miocene (23.03–5.33 Mya) [6,15,39]. Consistent with the general consensus on the evolutionary differentiation of the *Dryomys*, the molecular dating analysis using Bayesian Inference in this study estimated that *D. nitedula* and *D. laniger* diverged approximately 15.68 Mya, during the Middle Miocene (between 15.97 and 11.608 Mya).

The findings revealed high levels of intraspecific variation across the distribution range of *D. nitedula* populations examined in this study in line with the results of previous studies [6,15,18,19,20]. In the phylogenetic analyses, *D. nitedula* was found to have differentiated into three major clades (MC1, MC2, and MC3) two of which are further subdivided into multiple subclades (SC1 and SC2 in MC2 and SC3–SC5 in MC3). Moreover, it was determined that five different *D. nitedula* lineages (N1–N5) in Türkiye were grouped into these subclades (SC1–SC5), indicating high levels of genetic differentiation (K–2P distances ranged from 4.01% to 11.19%) that point out divergence at both the species and subspecies levels [50,51]. AMOVA revealed that the high genetic differentiation among *D. nitedula* lineages in Türkiye, as indicated by phylogenetic analyses, primarily arises from genetic differences with high percentage between the lineages (77.28%). This clearly demonstrated that the topologies obtained from ML and BI phylogenetic analyses were perfectly consistent with the geographical distribution pattern of the genetic structure of *D. nitedula* lineages in Türkiye. High bootstrap and BPP values supporting the nodes of the phylogenetic trees further strengthen this case.

### 4.1. Divergence of MC1

The initial differentiation events within *Dryomys nitedula* began around 8.82 Mya (node E) in the middle of Late Miocene (Tortonian stage), with the first major clade (MC1) to diverge being the one recognized as *Dryomys nitedula pictus* including Central Iran samples [19]. The suggested divergence time estimate for MC1 in the current study closely aligns with that of Mohammadi et al. [19], placing it around 7.8 mya (Late Miocene); diversification of this clade was likely influenced by orogenic events and aridification in Central Iran. MC1, designated *D. n. pictus* by Mohammadi et al. [19], which is also positioned at the base of the *D. nitedula* phylogeny in the current study, represents a deeply divergent lineage and should be considered a separate species, as suggested by Mohammadi et al. [19].

### 4.2. Divergence of MC2

The second major clade, MC2, which includes *Dryomys nitedula intermedius* and *Dryomys nitedula aspromontis* haplotypes from Italy (SC1), haplotypes from the Thrace region (N1 lineage, *Dryomys nitedula wingei*), the European part of Türkiye, and *Dryomys nitedula nitedula* haplotypes from Russia in Eastern Europe (SC2), was found to have diverged from MC3 approximately 7.15 Mya (node F), based on evolutionary dating analyses. Consistent with the current study, Mohammadi et al. [19] estimated the evolutionary divergence of this major clade at 5.5 Mya based on *CYTB* sequence variations. Compatible with this finding, Kankılıç et al. [6] dated its divergence to 5.88 Mya, based on variation in the *ND1* gene sequences. A genetic distance of approximately 9.3% was observed between the Russian haplogroups, which constitute the Eastern European haplogroup (EE), and the Western (WC) and Central Caucasian (CC) haplogroups based on *CYTB* sequences [18]. Similarly, in the current study, the genetic distance between the MC2, which includes haplotypes from Italy, Russia (EE), and Thrace, and the MC3, comprising WC and CC haplogroups from Russia and Caucasia, was found to be approximately 10.4%. Considering a mitochondrial mutation rate of 2% per million years [29,52], the genetic distance between the clades, roughly 4.65 million years according to Grigoryeva et al. [18] and 5.2 million years according to the current study, aligns well with the estimated evolutionary divergence times. The evolutionary divergence of MC2 roughly coincides with the onset of the Messinian Salinity Crisis, which triggered aridification and desertification in the Mediterranean basin [53,54,55]. Similarly, it has been proposed that the differentiation of the Thracian and Anatolian lineages during the Late Miocene may have been influenced either by geological processes that led to the formation of the Sea of Marmara and the Bosphorus and Dardanelles Straits (11.62–5.33 Mya), or by subsequent tectonic uplifts (5–6 Mya) in the region [6].

On the other hand, within the SC2 clade, geographical barriers such as the Kerch Strait, the Sea of Azov, the Lower Don River, the Kuban River, and the Manych Strait may have contributed to the genetic differentiation of the *D. n. nitedula* (Russian haplotypes in Eastern Europe, EE by Grigoryeva et al. [18]) from N1 (*D. n. wingei* in Thrace) lineage of Turkish *D. nitedula*. The genetic distance between these two lineages or haplogroups is approximately 1.14%, and based on BI analysis, it is estimated that they began diverging around 0.74 Mya (node S). This genetic distance value is consistent with those indicating intraspecific variation at the subspecies level [50,51]. Similarly to the results of current study, an evolutionary divergence between the northern European clade (containing Russian haplotypes) and the southern European clade (containing Turkish haplotypes) of *Spermophilus citellus* was detected at approximately 0.58 Mya [56]. This finding aligns well with the evolutionary divergence time observed between the N1 and EE clades in this study. According to Kryštufek et al. [56], the fragmentation of forests and steppe habitats led to the isolation of *S. citellus* clades from one another. A similar pattern of evolutionary differentiation was observed between the two clades of *Cricetus cricetus*, consisting of northern and southern haplogroups in Europe that diverged approximately 85,000 to 147,000 years ago [57]. Similarly to the genetic differentiation and the factors possibly driving it observed between the EE haplogroup and N1 lineages across their geographical distribution, the *Hyla orientalis* lineages also exhibit comparable patterns of genetic differentiation [58]. When considering the findings of this study alongside the distribution and differentiation patterns suggested for different taxa in previous research, as well as the factors influencing these patterns, we propose that the N1 and EE lineages of *D. nitedula* may have originated from two distinct glacial refugia around 0.74 million years ago. Specifically, the N1 lineage is likely to have originated in the Balkans, while the EE lineage may have come from the Carpathians or Eastern European refugia. As suggested by Dubey et al. [59] for *Crocidura* populations, the ancestral population in the Balkan refugium may have expanded as far north as the Danube River and as far south as the Dardanelles, the Sea of Marmara, and the Bosphorus. The ancestral population in the Carpathians and Eastern Europe may have extended southward to the Danube River and northward to the physical barriers of the Kerch Strait, the Sea of Azov, the Lower Don River, and the Kuban River during an interglacial period.

A divergence time of approximately 3.09 Mya was estimated for the split between SC1 and SC2 within MC2 (node M). The genetic distances between SC1 (*Dryomys nitedula intermedius* and *Dryomys nitedula aspromontis*) and SC2 (N1 lineage and haplogroup EE) ranged from approximately 4.45% to 7.12%. Consistent with the findings of this study, Mohammadi et al. [19] estimated that the evolutionary separation between the EE and Italian haplogroups occurred approximately 2.38 Mya, with genetic distances of 6.3% and 7.5% between these haplogroups. Italy serves as an important glacial refuge, with the Eastern Alps to the north acting as a significant geographic barrier between Italian lineages and other European lineages, including those from Eastern Europe [60]. Therefore, the formation processes of the Eastern Alps and Pleistocene climatic fluctuations are considered the primary factors driving the differentiation between the Italian lineages of *D. nitedula*, the EE haplogroup, and the N1 lineage. The geomorphological formation of the Eastern Alps began in the Oligocene (30–35 Mya), marked by the eastward growth of the mountain range, and was further accelerated by rock and surface uplift at the eastern end of the Alps during the Pliocene (5.33–2.58 Mya). The Pleistocene ice ages (2.58 Mya–11.7 Kyr ago) also had a significant impact on the landscape, carving cirques and extremely deep valleys, which were later filled [61]. *D. n. aspromontis* and *D. n. intermedius* are separated by vast plains between southern Italy and the eastern Alps. They are distributed in a philopatric manner, inhabiting beech-dominated forests as well as low-altitude mixed broadleaf and conifer forests [20]. Although previous studies suggested that these two subspecies, with a genetic distance of approximately 4.5%, diverged around 1 or 1.48 million years ago, the results of this study indicate that divergence actually began at the start of the Pleistocene, with a genetic distance between the subspecies consistent with the findings of earlier research [19,20].

### 4.3. Divergence of MC3

MC3 was the most diversified major clade, comprising *Dryomys nitedula* haplotypes from the Anatolian region of Türkiye, as well as previously reported subspecies from Russia, the Caucasus, and Iran, along with populations exhibiting significant genetic differences whose taxonomic status remains undetermined. Although MC3 was divided into more subclades (SC3, SC4, and SC5) than the other clades, the relative positions of these subclades and lineages under them within this major clade were not taxonomically ambiguous, as the geographic distributions of the studied populations were consistent with those of previously identified *D. nitedula* subspecies. The initial diversification within this clade occurred approximately 6 Mya (node G), at the onset of the Pliocene. This divergence time coincided with the Early Pliocene, a period marked by climatic cooling, fluvial incision, canyon formation, surface uplift, and significant tectonic and orogenic events [62].

The first diversified subclade, SC3, was positioned at the base of MC3. SC3 contained N2 (Black Sea) and N3 (Anatolia) lineages of *Dryomys nitedula*. The haplotypes forming N2 lineage were distributed over a wide area, starting from the coastal region of Artvin in the eastern part of the Eastern Black Sea and extending to the Trabzon–Giresun border in the west of this region. The eastern limit of the distribution area of this lineage extends to the inner regions of Artvin. The distribution of haplotypes forming the N3 lineage starts in Giresun in the north and spans a vast area, extending through suitable habitats surrounding central Anatolia to the east, south, and west of Türkiye. The west side of the Anatolian Diagonal is the easternmost distribution limit of this lineage. The subspecies *Dryomys nitedula phrygius*, originally described from Muratdağı (Uşak) in western Anatolia, is considered to be widely distributed across Anatolia, including western Anatolia, the Black Sea region, the Mediterranean, and central Anatolia [1]. However, the findings reveal the presence of two distinct lineages: one originating in the area southeast of the Dardanelles, including the Sea of Marmara and the Bosphorus in western Anatolia, encircling central Anatolia, extending southward and northward, and reaching the western side of the Anatolian Diagonal; the other inhabiting the coastal areas of the Eastern Black Sea region (N2). The differentiation between the N2 and N3 lineages aligns with the findings of previous studies based on allozyme, mitochondrial DNA, and combinations of mitochondrial and nuclear DNA [4,6,15]. The geographical boundary of the distribution areas of the N2 and N3 lineages lies between Bulancak (Giresun) and Maçka (Trabzon) in the Eastern Black Sea region. This area spans approximately 120 km and is home to numerous large and small streams, including Batlama Stream, Aksu Stream, Yağlı Stream, Özlüce Stream, Doğankent Stream, Görele Stream, Fol Stream, İskefiye Stream, Söğütlü Stream, and Değirmendere. It is anticipated that these streams may have played a role in the allopatric differentiation of the two lineages. From a taxonomic perspective, the N3 lineage, with its distribution limits outlined in detail, confirms the existence of the subspecies *D. n. phrygius*. Kryštufek and Vohralík [1] classified *Dryomys nitedula* populations in northeastern Türkiye, including those from Rize, as subspecies *Dryomys nitedula tichomirowi*. However, in this study, haplotypes from Maçka (Trabzon), Rize, as well as Hopa and Arhavi on the coastal side of Artvin in the Eastern Black Sea region, formed a distinct lineage (N3), which is closely related to N2 (*D. n. phrygius*), instead *D. n. tichomirowi* or N4 lineages within the SC4. There is a genetic distance of 8.59% and 8.98% between the N2 and N4 lineages of Turkish *D. nitedula*, and between N2 and *D. n. tichomirowi* [19], respectively, both of which were found to be quite distinct lineages or haplogroups by BI and ML analyses in the current study. The N4 lineage, grouped with *D. n. tichomirowi*, consists of haplotypes from the interior of Artvin, as well as Kars and Ardahan, located in the northern part of the eastern Anatolia region. The Çoruh River Canyon likely forms the geographical boundary between the distribution areas of the N2 and N4 lineages of Turkish *D. nitedula*, contributing to the allopatric differentiation of these lineages. In this case, the N2 lineage, which is genetically distinct from both the N3 and N4 lineages, represents a potential new subspecies of *D. nitedula* found in areas near the coastal regions of the Eastern Black Sea in Türkiye. Evolutionary divergence of the N2 and N3 lineages of Turkish *D. nitedula* date back to the beginning of the Pliocene (2.56 Mya, node O), with a genetic distance of 4%, supporting subspecies level of divergence [50,51]. This divergence age was congruent with the Pliocene–Pleistocene climate transitions [62]. Given the genetic distances between the N2 and N3 lineages and evolutionary divergence time, it can be inferred that the topographic structure of the region, along with climatic fluctuations during the Pleistocene, played a significant role in the differentiation of this potential new subspecies [63,64]. Supporting this, Helvacı et al. [65] suggested that a secondary differentiation of *Glis glis* occurred within the eastern region, including the central Black Sea coast (Ordu). This population was found to be distinct from the Colchic localities (Trabzon, Rize, Artvin), which represent the main range of the potential new subspecies (N2). On the other hand, there is an approximate 8.59% genetic distance between the N2 and N4 lineages. Furthermore, the SC3 and SC4 clades, which encompass these two lineages, began to diverge evolutionarily during the Late Miocene to Early Pliocene period, roughly 6 Mya. In a previous study based on mitochondrial DNA *ND1* sequences, Kankılıç et al. [6] estimated a divergence time of 3.80 million years between the Black Sea and Şavşat haplogroups of *D. nitedula* on the west and east sides of the Çoruh River Canyon. Similarly to the divergence of the N2 and N4 lineages of *D. nitedula* in the current study, the influence of the Çoruh River Canyon was highlighted in the divergence of lineages within the *Phonochorion* bush cricket genus, which is endemic to the Western Lesser Caucasus hotspot and distributed in the same region. It was estimated that the lineages on the east and west sides of the canyon diverged approximately 5.849 Mya, with a range between 3.727 and 8.589 Mya [66]. River incision and the formation of the canyon in the area during the Late Miocene (Messinian) and Pliocene periods [55,67,68,69] may have separated the N2 and N4 lineages of *D. nitedula*, as suggested by Sağlam et al. [66] for differentiation within the genus *Phonochorion*. In this case, it is reasonable to conclude that lineage N2, genetically distinct from both N3 and N4, represents a potential new subspecies of *D. nitedula* that diverged from these lineages during the Pliocene–Pleistocene transition in the area extending from the coastal side of Artvin to the border Maçka (Trabzon) and Bulancak (Giresun) in the northeastern Black Sea region. Undoubtedly, detailed biometric and molecular analyses of the populations comprising the N2 lineage of *Dryomys nitedula*, which are considered a potential new subspecies, will provide a clearer understanding of their taxonomic status.

SC4 contains WC (West Caucasian haplotypes or *Dryomys nitedula heptneri* from Western Caucasus), CC (*D. n. ognevi* from Central Caucasus), *D. n. tichomirowi* from Iran and N4 lineage from Türkiye. The first diverged haplogroup was *D. n. heptneri* with a split age 4.64 Mya (node J). The genetic distance between WC and other haplogroups within SC4 was 7.19%. This split age corresponds to the Early Middle Pliocene. Mohammadi et al. [19] determined that the genetic differentiation between WC and CC began approximately 3.08 Mya, with a genetic distance of 7.2%, which is consistent with the results of the current study. Grigoryeva et al. [18] reported a genetic distance of 6% between these haplogroups. The separation of the WC and CC haplogroups may be attributed to an uplift in the eastern part of the Greater Caucasus that occurred from the Late Miocene through the Pliocene and Early Pleistocene (7–2 Mya). This uplift caused the Central and Eastern Caucasus to rise by 1.5 to 2 km, with elevations in the Elbrus region reaching 3750 m [70]. Interestingly, haplogroup CC (*Dryomys nitedula ognevi*) includes haplotypes of *D. nitedula* from the Elbrus region in the Central Caucasus, while WC (*D. n. heptneri*) contains haplotypes from west Caucasia [18]. We suggest that the Pliocene cooling, along with the elevation differences between the Western and Central–Eastern Caucasus, as well as geographic or reproductive isolation [18], may have contributed to the differentiation of both WC (*D. n. heptneri*) and CC (*D. n. ognevi*). Mohammadi et al. [19] suggested that subspecies differentiation in the Caucasus may have been caused by forest fragmentation during the Pliocene and pre-Pleistocene periods, leading to interrupted distributions and isolation. Additionally, they proposed that remaining populations in the Zagros Mountains may have been isolated due to their location between the desert regions of Azerbaijan and Iran.

Physical barriers, such as the Greater Caucasus Mountains and the Transcaucasian Foreland, are believed to contribute to the isolation of the haplogroup CC (*D. n. ognevi* from Central Caucasus), N4 lineage of Turkish *Dryomys nitedula* and *Dryomys nitedula tichomirowi* from Iran within SC4. Haplogroup CC diverged from the N4 lineage of Turkish *D. nitedula* plus *D. n. tichomirowi* from Iran approximately 3.56 Mya (node L), with a genetic distance of 6.94% between CC and both N4 plus *D. n tichomirowi*. Also, the N4 lineage and *D. n. tichomirowi* diverged from each other approximately 1.77 Mya (node R), with a genetic distance of 4.72%. The divergence times and genetic distances for the split of *D. nitedula* haplogroups in the Caucasus are consistent with the findings of Mohammadi et al. [19]. These diversification events coincide with the Early Late Pliocene cooling and Pleistocene glaciations [62], which may have confined *D. nitedula* populations to Caucasian glacial refugia, as suggested by Mohammadi et al. [19]. Considering the genetic diversity levels of haplogroups, lineages, or subspecies within SC4, it is clear that the N4 lineage exhibits the highest values in terms of nucleotide diversity. Moreover, this lineage has 3.5 times higher nucleotide diversity than haplogroup WC, 2.86 times higher than haplogroup CC, 4.82 times higher than *D. n. heptneri*, and 25 times higher than *D. n. tichomirowi*. The number of individuals in haplogroups influences this pattern, but still ancestral populations typically have high nucleotide diversity, while descendant populations often show lower diversity due to evolutionary mechanisms like genetic bottlenecks or founder effects. The exceptionally high nucleotide diversity observed in the N4 lineage compared to other haplogroups in the Caucasus suggests the potential existence of a glacial refuge for *D. nitedula* near Sarıkamış (Kars), at elevations over 2000 m, in northern eastern Anatolia. It is clear that stronger evidence, including additional molecular markers and a larger sample size, is needed to clarify this situation more accurately. The high genetic distances of approximately 7%, indicating species-level divergence, between the haplogroups grouped together in SC4 and distributed across the Caucasus, along with the evolutionary divergence events that occurred from the beginning of the Pliocene to the middle of the Pleistocene within this subclade, suggest that these haplogroups should be considered a species complex. A similar conclusion was drawn by Mohammadi et al. [19] for haplogroups in the Western Palearctic.

SC5 diverged from SC4 around 5.45 Mya during the Early Pliocene (node H). This subclade includes haplotypes of *Dryomys nitedula* from the southeastern Anatolia region of Türkiye, which were previously classified as *Dryomys nitedula pictus*, as well as the subspecies *Dryomys nitedula kurdistanicus* from Zagros and *Dryomys nitedula bilkjewiczi* from Kopet Dagh in Iran. *Dryomys nitedula bilkjewiczi* represents the earliest diverging clade within this subclade, with a split age of 3.57 Mya during the Late Pliocene (node K). This subspecies exhibits a genetic distance of approximately 5.47% from *D. n. kurdistanicus* and the N5 lineage. Supporting the timing and genetic distances of this evolutionary separation, Mohammadi et al. [19] suggested that *D. n. bilkjewiczi* from Kopet Dagh and *D. n. kurdistanicus* from the Zagros may have diverged during the Late Pliocene to Early Pleistocene (HPD: 0.79–2.95 Mya, K–2P: 5.9%). This divergence likely occurred in the Zagros and Kopet Dag refugia, with the fragmentation of ancestral populations into distinct refugia [19]. The geographical distribution of specimens from Türkiye in this subclade (N5 lineage), previously recorded only from Hakkari and classified as *Dryomys pictus* in an earlier study [22], then *D. n. pictus* [1], extends from Erzurum in the north to Elazığ in the west and Hakkari in the southeastern. Genetic differentiation between N5 lineage and *D. n. kurdistanicus* from the Zagros is estimated to have begun around 2.12 Mya, with a genetic distance of 4.90% (node Q). This timing coincides with the Pliocene–Pleistocene transition. Possibly, the Çataksuyu River (also known as Çığlı Suyu or Zap Suyu) located in the southern part of the N5 lineage’s distribution range, may have acted as a geographic barrier, isolating this lineage from *D. n. kurdistanicus*. To the north and west, the distribution of N5 lineage may be constrained by the Anatolian Diagonal, a mountain range complex stretching from the Erzurum–Kars plateau in the north to the Amanus and Central Taurus Mountains in the south. Thus, the Anatolian Diagonal appears to be the primary factor limiting the western expansion of the N5 lineage and isolating it from the N3 (*D. n. phrygius*) lineage. Numerous studies on various organisms (including the Orthoptera group, *Andricus caputmedusae*, *Spermophilus xanthoprymnus*, *Ommatotriton vittatus*, *Microtus guentheri*, *Arvicola amphibius*, *Elaphe sauromates*, and *E. urartica* sp. nov.) have documented the Anatolian Diagonal as a key factor driving intraspecific differentiation, acting as both an ecological and geographical barrier [71,72,73,74,75,76,77]. The distributions of the N5 and N3 lineages are confined between Elazığ, located at the westernmost extent of the N5 lineage range, and Malatya, at the easternmost extent of the N3 lineage range. In the Malatya basin, evidence suggests the existence of a paleo-lake system that reached its maximum extent around 4 Mya [78]. The Euphrates River in this basin is linked to a series of lake basins extending across Elazığ, Malatya, and Adıyaman [67,79]. This ancient water system may have played a significant role in the differentiation of the N5 and N3 lineages. On the other hand, the distributions of the N5 and N4 lineages are limited between Erzurum, located at the northernmost extent of the N5 lineage range, and Kars at the southernmost extent of the N4 lineage range. Samples collected from Erzurum, the northernmost point of the N5 lineage distribution, were obtained from the southern part of the Karasu River. Meanwhile, samples from the southernmost part of the N4 lineage distribution were collected from the northern part of the Aras River basin. This highlights the influence of river systems, such as the Euphrates River to the west and the Karasu, Murat, and Aras River basins to the north, on the differentiation of *D. nitedula* lineages in the region. In addition to the impact of Anatolian Diagonal, these rivers may have acted as vicariance agents, driving the genetic differentiation between the N5, N4, and N3 lineages.

In previous studies, *Dryomys nitedula* samples from Hakkari, located at the southeasternmost extent of the N5 lineage distribution, were first classified as the species *Dryomys pictus* [22] and then the subspecies *Dryomys nitedula pictus* [1]. However, while the two samples collected south of Çataksuyu clustered with other N5 haplotypes from Türkiye in the ML and BI trees, haplotype network analysis indicated that these samples were more closely related to the subspecies *Dryomys nitedula kurdistanicus* from Zagros. Given the distribution of *D. n. pictus* in Iran and the geographical barriers—such as deserts and salt plains on the Iranian Plateau, and the Elbrus and Zagros mountains—that isolate it from other subspecies, it seems questionable to classify *D. nitedula* haplotypes from the eastern Anatolian region of Türkiye, including Hakkari, as *D. n. pictus*. The other haplotypes that make up lineage N5, along with the Hakkari haplotypes, are more closely related to *D. n. kurdistanicus* than to *D. n. pictus*. Additionally, a genetic distance of approximately 12.92% exists between the N5 lineage and *D. n. pictus*, suggesting that they represent two possible distinct biological species. Therefore, as suggested by Mohammadi et al. [19], the taxonomic status of lineage N5, *D. n. kurdistanicus*, *D. n. bilkjewiczi*, and *D. n. pictus* should be reassessed using additional sampling and molecular markers.

### 4.4. Divergence of Dryomys laniger

Populations of *Dryomys laniger* were split into two lineages (L1 and L2 or *Dryomys anatolicus*) around 2.94 Mya during the Late Pliocene (node N). There is a genetic distance of 5.47% between the two lineages. L1 includes haplotypes from Niğde (Bolkar Mountains, including Demirkazık, Madenköy, and Meydan Plateau) and Antalya (Çığlıkara) at altitudes of 1700–1780 m above sea level. The L2 lineage (*D. anatolicus*) consists of haplotypes from Kahramanmaraş (Göksun) and Malatya (Darende), at altitudes of 1452 m above sea level. The Göksun basin, which is located in the distribution range of these two lineages and has a high level of tectonic activity, may have acted as a physical barrier contributing to their differentiation. The Sürgü Fault Zone (SFZ), extending from Göksun (Kahramanmaraş) in the west to Çelikhan (Adıyaman) in the east of southeastern Anatolia, experienced westward displacement during the Pliocene. The southern block of the SFZ underwent a sudden elevation change and movement towards the alluvial plain of the Göksun Basin [80,81,82]. Therefore, it is believed that tectonic activities, specifically the uplift of the southern block of the SFZ during the Pliocene–Pleistocene, may have led to the separation of the L1 lineage from the L2 lineage (*D. anatolicus*) of *D. laniger*. Based on mitochondrial and nuclear DNA sequences, as well as morphological aspects, Çetintaş et al. [26] identified two lineages of *D. laniger*: Eastern and Western, with a genetic distance of 7%. They assigned the Eastern lineage to a new *Dryomys* species as *Dryomys anatolicus*. The results of the current study support the taxonomic distinction between the two lineages of *D. laniger* proposed by [23,26]. Although this study’s results are consistent with this situation, it is believed that a larger sample size and the use of additional molecular markers would likely provide more taxonomically clear results.

## 5. Conclusions

Anatolia, located between the eastern part of the European continent and the Near East, and home to species of Balkan and Caucasian origin, is believed to have served as a potential source and refuge for intraspecific diversity in Europe during the glacial period. However, the effects of climatic, geological, ecological, and geographical factors in Anatolia on the species inhabiting this region have not been studied in detail [27,28,29]. In this context, the intraspecific variability and the factors thought to contribute to it, observed in two *Dryomys* species (*Dryomys nitedula* and *Dryomys laniger*), have been discussed in detail in the current study. The results obtained revealed that *Dryomys nitedula* populations in Türkiye have differentiated into five distinct lineages (N1–N5), exhibiting significant genetic differences. It is thought that their evolution has been influenced by the varied topographic features and fluctuating climatic conditions of Anatolia. Among these lineages, the N2 lineage, found in regions near the Eastern Black Sea coast, is believed to represent a new subspecies. The genetic diversity within each lineage of *D. nitedula* in Anatolia, as well as the total genetic diversity across all lineages, is notably high when compared to the haplogroups or subspecies identified by previous studies from adjacent regions. Evidence from mitochondrial DNA *CYTB* gene sequences suggests that Anatolia may have served as a refuge for *D. nitedula* during the Pleistocene glacial period. Due to Türkiye’s geographical location, this study on *D. nitedula* populations, using mitochondrial DNA *CYTB* sequences, provides a clearer understanding of the taxonomic status of *D. nitedula* populations in Europe and the Near East by integrating both current and past findings. However, it is suggested that using a broader range of molecular markers would be more effective in definitively determining the taxonomic status of populations that exhibit genetic differences at the species level [18,19,20]. In the endemic *Dryomys laniger*, which has a more restricted distribution, genetic variability shaped by past and present environmental factors revealed the presence of two main lineages (L1 and L2 or *Dryomys anatolicus*). To make a more accurate taxonomic assessment of *D. laniger* and also *D. anatolicus*, a larger number of samples is needed, and the distribution limits should be more clearly defined.

## Figures and Tables

**Table 2:** Genetic diversity statistics for the lineages of *Dryomys*. N: sample size, S: number of segregating sites, NH: number of haplotypes, H: haplotype diversity with standard deviation, π: nucleotide diversity with standard deviation.

Taxon/Clade		N	S	NH	H ± SD	π ± SD
*D. nitedula* (Türkiye)	All	88	218	47	0.981 ± 0.005	0.06928 ± 0.00190
	N1	18	23	10	0.902 ±0.050	0.00789 ± 0.00078
	N2	17	57	9	0.912 ±0.042	0.00968 ± 0.00454
	N3	23	77	14	0.953 ± 0.025	0.02391 ± 0.00152
	N4	13	43	8	0.897 ± 0.067	0.01627 ± 0.00455
	N5	17	56	6	0.816 ± 0.061	0.01519 ± 0.00558
	N2 + N3 + N4 + N5	70	189	37	0.976 ± 0.006	0.05910 ± 0.00222
*D. n. nitedula* (Russia)	EE	10	12	9	0.978 ± 0.054	0.00579 ± 0.00082
*D. n. heptneri* (Caucasia)	WC	5	10	5	1.000 ± 0.01600	0.00466 ± 0.00076
*D. n. ognevi* (Caucasia)	CC	3	8	3	1.000 ± 0.07407	0.00567 ± 0.00189
*D. n. aspromontis* (Italy)		8	3	3	0.714 ± 0.123	0.00305 ± 0.00080
*D. n. intermedius* (Italy)		7	1	2	0.571 ± 0.119	0.00135 ± 0.00028
*D. n. pictus* (Iran)		11	18	6	0.873 ± 0.071	0.00759 ± 0.00110
*D. n. tichomirowi* (Iran)		6	1	2	0.600 ± 0.129	0.00064 ± 0.00014
*D. n. heptneri* (Russia)		2	3	2	1.000 ± 0.500	0.00337 ± 0.00169
*D. n. kurdistanicus* (Iran)		14	13	7	0.879 ± 0.058	0.00401 ± 0.00086
*D. n. bilkjewiczi* (Iran)		14	22	8	0.857 ± 0.077	0.00497 ± 0.00108
*D. laniger* (Türkiye)	All	22	64	8	0.792 ± 0.069	0.02714 ± 0.00369
	L1	15	21	6	0.648 ± 0.134	0.00567 ± 0.00127
	L2	7	9	2	0.476 ± 0.171	0.00455 ± 0.00164

**Table 3:** Genetic distances (K–2P) between the lineages and subspecies of *D. nitedula*. The lower part of the diagonal displays genetic distances, while the upper part shows the standard deviations.

Clade	1	2	3	4	5	6	7	8	9	10	11	12	13	14
1. N1		0.0104	0.0112	0.0113	0.0111	0.0025	0.0111	0.0120	0.0095	0.0116	0.0130	0.0103	0.0113	0.0112
2. N3	0.0983		0.0053	0.0088	0.0091	0.0108	0.0088	0.0105	0.0157	0.0164	0.0115	0.0085	0.0101	0.0085
3. N2	0.1045	0.0401		0.0091	0.0097	0.0120	0.0097	0.0105	0.0158	0.0171	0.0125	0.0092	0.0103	0.0095
4. N4	0.1119	0.0876	0.0859		0.0093	0.0114	0.0091	0.0082	0.0180	0.0190	0.0120	0.0054	0.0105	0.0091
5. N5	0.1097	0.0874	0.0904	0.0932		0.0119	0.0093	0.0101	0.0143	0.0149	0.0119	0.0094	0.0066	0.0067
6. *D. n. nitedula*	0.0115	0.0993	0.1070	0.1085	0.1127		0.0117	0.0121	0.0149	0.0177	0.0139	0.0104	0.0124	0.0117
7. *D. n. heptneri*	0.0987	0.0768	0.0812	0.0780	0.0830	0.1005		0.0093	0.0160	0.0174	0.0118	0.0074	0.0102	0.0089
8. *D. n. ognevi*	0.1173	0.1008	0.0960	0.0686	0.0902	0.1138	0.0730		0.0185	0.0202	0.0134	0.0079	0.0110	0.0100
9. *D. n. aspromontis*	0.0445	0.1011	0.0964	0.1233	0.0826	0.0562	0.0934	0.1187		0.0101	0.0189	0.0164	0.0158	0.0158
10. *D. n. intermedius*	0.0568	0.0985	0.0997	0.1289	0.0858	0.0712	0.1048	0.1299	0.0427		0.0202	0.0181	0.0157	0.0171
11. *D. n. pictus*	0.1348	0.1185	0.1268	0.1258	0.1292	0.1412	0.1213	0.1395	0.1247	0.1340		0.0119	0.0129	0.0123
12. *D. n. tichomirowi*	0.1028	0.0867	0.0898	0.0472	0.0970	0.1014	0.0616	0.0707	0.1124	0.1254	0.1302		0.0100	0.0087
13. *D. n. kurdistanicus*	0.1112	0.0937	0.0899	0.0989	0.0490	0.1183	0.0886	0.0964	0.0954	0.0839	0.1361	0.0997		0.0078
14. *D. n. bilkjewiczi*	0.1018	0.0761	0.0811	0.0801	0.0523	0.1026	0.0696	0.0827	0.0924	0.1022	0.1242	0.0780	0.0575	

## Data Availability

Materials (skins, tissues, and skulls) used in this study are available in Ankara University Mammalian Research Collection (AUMAC; https://mammalia.ankara.edu.tr/, 10 January 2024) and Mammalian Tissue Collection in Department of Biology, Faculty of Science, Ankara University, Ankara, Türkiye. Mitochondrial DNA cytochrome *b* sequence data are available at GenBank nucleotide database (https://www.ncbi.nlm.nih.gov/nuccore, 4 March 2025).

## Abbreviations

The following abbreviations are used in this manuscript:

*CYTB*	Cytochrome b
MC1	Major clade 1
MC2	Major clade 2
MC3	Major clade 3
SC1	Subclade 1
SC2	Subclade 2
SC3	Subclade 3
SC4	Subclade 4
SC5	Subclade 5
N1	Thracian lineage of *D. nitedula*
N2	Black Sea lineage of *D. nitedula*
N3	West and central Anatolia lineage of *D. nitedula*
N4	Northeastern Anatolia lineage of *D. nitedula*
N5	Eastern Anatolia lineage of *D. nitedula*
L1	Western lineage of *D. laniger*
L2	Eastern lineage of *D. laniger*
CTAB	Cetyltrimethylammonium bromide DNA isolation method
PCR	Polymerase chain reaction
TAE	Tris–Acetate–EDTA buffer
MCMC	Markov Chain Monte Carlo algorithm
ML	Maximum Likelihood
BI	Bayesian Inference
BIC	Bayesian Information Criterion
AICc	Corrected Akaike Information Criterion
TN93 + G + I	Tamura Nei 1993 nucleotide substitution model with Gamma distribution Invariant sites
HKY + G + I	Hasegawa–Kishino–Yano 1985 nucleotide substitution model with Gamma distribution Invariant
Mya	Million years ago
K–2P	Kimura–2-Parameter
WC	West Caucasian haplogroup
CC	Central Caucasian haplogroup
EE	Eastern Europe haplogroup

## Appendix A

**Table A1:** Localities and corresponding coordinates of sample collection sites of *Dryomys* in Türkiye and Bulgaria.

Map Number	Species	Lineage	Locality	Sample Size	Coordinates	
1	*D. nitedula*	N1	Bulgaria	1	42°41′51″ N	23°19′18″ E
2	*D. nitedula*	N1	Edirne	11	40°46′33″ N	26°26′22″ E
3	*D. nitedula*	N1	Edirne	2	41°39′23″ N	26°33′19″ E
4	*D. nitedula*	N1	Kumbağ (Tekirdağ)	1	40°52′25″ N	27°25′18″ E
5	*D. nitedula*	N1	Gelibolu (Çanakkale)	3	40°25′3″ N	26°40′19″ E
6	*D. nitedula*	N3	Çan (Çanakkale)	4	39°58′36″ N	27°5′34″ E
7	*D. nitedula*	N3	Abant (Bolu)	2	40°34′41″ N	31°16′30″ E
8	*D. nitedula*	N3	Bursa	5	40°8′37″ N	29°5′57″ E
9	*D. nitedula*	N3	Eber (Afyon)	1	38°36′19″ N	31°7′30″ E
10	*D. nitedula*	N3	Madenköy (Niğde)	2	37°26′43″ N	34°37′30″ E
11	*D. nitedula*	N3	Pınarbaşı (Kayseri)	1	38°42′13″ N	36°26′21″ E
12	*D. nitedula*	N3	Elmalı (Antalya)	2	36°43′2″ N	29°52′24″ E
13	*D. nitedula*	N3	Çakallı (Samsun)	1	41°8′17″ N	36°7′1″ E
14	*D. nitedula*	N3	Bulancak (Giresun)	1	40°49′54″ N	38°14′36″ E
15	*D. nitedula*	N3	Malatya	4	38°32′39″ N	37°30′58″ E
16	*D. nitedula*	N2	Çat (Rize)	3	40°52′3″ N	40°56′5″ E
17	*D. nitedula*	N2	Çamlıhemşin (Rize)	4	41°3′4″ N	41°1′1″ E
18	*D. nitedula*	N2	Maçka (Trabzon)	1	40°41′33″ N	39°39′39″ E
19	*D. nitedula*	N2	Arhavi (Artvin)	1	41°18′13″ N	41°24′34″ E
20	*D. nitedula*	N2	Hopa (Artvin)	8	41°23′13″ N	41°30′34″ E
21	*D. nitedula*	N4	Ardanuç (Artvin)	4	41°7′9″ N	42°7′21″ E
22	*D. nitedula*	N4	Şavşat (Artvin)	5	41°22′29″ N	42°22′54″ E
23	*D. nitedula*	N4	Posof (Ardahan)	1	41°29′58″ N	42°45′11″ E
24	*D. nitedula*	N4	Kars	3	40°17′26″ N	42°36′19″ E
25	*D. nitedula*	N5	Erzurum	5	39°55′53″ N	40°43′42″ E
26	*D. nitedula*	N5	Tatvan (Bitlis)	7	38°27′26″ N	42°18′55″ E
27	*D. nitedula*	N5	Elazığ	3	38°36′52″ N	39°32′49″ E
28	*D. nitedula*	N5	Hakkari	2	37°36′45″ N	43°51′15″ E
29	*D. laniger*	L1	Elmalı (Antalya)	1	36°46′33″ N	29°55′11″ E
30	*D. laniger*	L1	Madenköy (Niğde)	5	37°26′17″ N	34°36′55″ E
31	*D. laniger*	L1	Bolkar (Niğde)	9	37°24′40″ N	34°34′10″ E
32	*D. laniger*	L2	Kahramanmaraş	5	37°55′49″ N	36°35′53″ E
33	*D. laniger*	L2	Malatya	2	38°36′52″ N	37°34′45″ E

**Table A2:** Haplotype list of *Dryomys* species from Türkiye and Bulgaria, and outgroup samples downloaded from GenBank. H: haplotype number, F: frequency, MN: map number.

Taxon/Clade	H	F	Locality and Voucher Number/ Accession No. of GenBank Samples	MN	Accession No./Reference
*D. n. wingei* (N1)	1	1	Bulgaria (11b)	1	PV216972
*D. n. wingei* (N1)	2	5	Edirne (5156, 5160, 5609, 5612, 7163)	2	PV216973
*D. n. wingei* (N1)	3	2	Edirne (5157, 5162)	2	PV216974
*D. n. wingei* (N1)	4	1	Edirne (5158)	2	PV216975
*D. n. wingei* (N1)	5	1	Edirne (5159)	2	PV216976
*D. n. wingei* (N1)	6	1	Edirne (5161)	2	PV216977
*D. n. wingei* (N1)	7	1	Edirne (5163)	2	PV216978
*D. n. wingei* (N1)	8	2	Edirne (5543, 7162)	3	PV216979
*D. n. wingei* (N1)	9	3	Kumbağ–Tekirdağ (7207), Gelibolu–Çanakkale (7219, 7164)	4, 5	PV216980
*D. n. wingei* (N1)	10	1	Gelibolu–Çanakkale (7201)	5	PV216981
*D. n. phrygius* (N3)	11	2	Çan–Çanakkale (7262, 7274)	6	PV216982
*D. n. phrygius* (N3)	12	2	Çan–Çanakkale (7264, 7285)	6	PV216983
*D. n. phrygius* (N3)	13	2	Abant–Bolu (7130, 7186)	7	PV216984
*D. n. phrygius* (N3)	14	1	Bursa (7419)	8	PV216985
*D. n. phrygius* (N3)	15	2	Bursa (7440, 7442)	8	PV216986
*D. n. phrygius* (N3)	16	1	Bursa (7441)	8	PV216987
*D. n. phrygius* (N3)	17	1	Bursa (7443)	8	PV216988
*D. n. phrygius* (N3)	18	1	Eber–Afyon (5589)	9	PV216989
*D. n. phrygius* (N3)	19	2	Madenköy–Niğde (2322, 2323)	10	PV216990
*D. n. phrygius* (N3)	20	1	Pınarbaşı–Kayseri (2929)	11	PV216991
*D. n. phrygius* (N3)	21	2	Elmalı–Antalya (5749, 5756)	12	PV216992
*D. n. phrygius* (N3)	22	1	Çakallı–Samsun (5357)	13	PV216993
*D. n. phrygius* (N3)	23	1	Bulancak–Giresun (7208)	14	PV216994
*D. n. phrygius* (N3)	24	4	Malatya (7470, 7471, 7472, 7473)	15	PV216995
A potential new taxon (N2)	25	2	Çat–Rize (5358, 5360)	16	PV216996
A potential new taxon (N2)	26	1	Çat–Rize (5361)	16	PV216997
A potential new taxon (N2)	27	1	Çamlıhemşin–Rize (5392)	17	PV216998
A potential new taxon (N2)	28	2	Çamlıhemşin–Rize (5400, 5404)	17	PV216999
A potential new taxon (N2)	29	1	Çamlıhemşin–Rize (5427)	17	PV217000
A potential new taxon (N2)	30	1	Maçka–Trabzon (7282)	18	PV217001
A potential new taxon (N2)	31	2	Arhavi–Artvin (7381), Hopa–Artvin (7338)	19, 20	PV217002
A potential new taxon (N2)	32	3	Hopa–Artvin (7339, 7379), Cankurtaran–Artvin (5713)	20	PV217003
A potential new taxon (N2)	33	4	Hopa–Artvin (7340), Cankurtaran–Artvin (7341, 7342, 7378)	20	PV217004
*D. n. tichomirowi* (N4)	34	4	Ardanuç–Artvin (7374, 7375, 7376, 7377)	21	PV217005
*D. n. tichomirowi* (N4)	35	2	Şavşat–Artvin (5712, 7371)	22	PV217006
*D. n. tichomirowi* (N4)	36	1	Şavşat–Artvin (7368)	22	PV217007
*D. n. tichomirowi* (N4)	37	1	Şavşat–Artvin (7372)	22	PV217008
*D. n. tichomirowi* (N4)	38	1	Şavşat–Artvin (7373)	22	PV217009
*D. n. tichomirowi* (N4)	39	1	Posof–Ardahan (7345)	23	PV217010
*D. n. tichomirowi* (N4)	40	2	Kars (7474, 7476)	24	PV217011
*D. n. tichomirowi* (N4)	41	1	Kars (7475)	24	PV217012
*D. n. pictus* (N5)	42	1	Erzurum (7478)	25	PV217013
*D. n. pictus* (N5)	43	4	Erzurum (7480, 7481, 7482, 7483)	25	PV217014
*D. n. pictus* (N5)	44	6	Tatvan–Bitlis (7263, 7265, 7276, 7287, 7288, 7289)	26	PV217015
*D. n. pictus* (N5)	45	1	Tatvan–Bitlis (7268)	26	PV217016
*D. n. pictus* (N5)	46	3	Elazığ (7277, 7290, 7291)	27	PV217017
*D. n. pictus* (N5)	47	2	Hakkari (7266, 7273)	28	PV217018
*D. n. nitedula* (EE)	48	1	Belorussia (KJ739693)	–	[18]
*D. n. nitedula* (EE)	49	1	Russia (KF699220)	–	[18]
*D. n. nitedula* (EE)	50	1	Russia (KF699221)	–	[18]
*D. n. nitedula* (EE)	51	1	Russia (KF699223)	–	[18]
*D. n. nitedula* (EE)	52	1	Russia (KF699226)	–	[18]
*D. n. nitedula* (EE)	53	1	Russia (KJ739694)	–	[18]
*D. n. nitedula* (EE)	54	1	Russia (KJ739695)	–	[18]
*D. n. nitedula* (EE)	55	1	Russia (KJ739696)	–	[18]
*D. n. nitedula* (EE)	56	1	Russia (KJ739697)	–	[18]
*D. n. nitedula* (EE)	57	1	Russia (KJ739698)	–	[18]
*D. n. heptneri* WC	58	1	Russia (KJ739699)	–	[18]
*D. n. heptneri* WC	59	1	Russia (KJ739700)	–	[18]
*D. n. heptneri* WC	60	2	Russia (KJ739701, KF699218)	–	[18]
*D. n. heptneri* WC	61	1	Russia (KJ739702)	–	[18]
*D. n. heptneri* WC	62	1	Russia (AJ225116)	–	[18]
*D. n. ognevi* (CC)	63	1	Russia (KJ739703)	–	[18]
*D. n. ognevi* (CC)	64	1	Russia (KJ739704)	–	[18]
*D. n. ognevi* (CC)	65	1	Russia 8KJ739705)	–	[18]
*D. n. aspromontis*	66	4	S Italy (MH671555, MH671556, MH671557, MH671558)	–	[20]
*D. n. aspromontis*	67	2	S Italy, (MH671559, MH671560)	–	[20]
*D. n. aspromontis*	68	2	S Italy, (MH671561 MH671562)	–	[20]
*D. n. intermedius*	69	4	NE Italy, (MH671563, MH671564, MH671565, MH671566)	–	[20]
*D. n. intermedius*	70	3	NE Italy (MH671567, MH671568, MH671569)	–	[20]
*D. n. pictus*	71	1	Yazd–Iran (MT559843)	–	[19]
*D. n. pictus*	72	2	Yazd–Iran (MT559842, MT559838)	–	[19]
*D. n. pictus*	73	1	Yazd–Iran (MT559841)	–	[19]
*D. n. pictus*	74	3	Yazd–Iran (MT559840, MT559839, MT559836)	–	[19]
*D. n. pictus*	75	1	Yazd–Iran (MT559837)	–	[19]
*D. n. pictus*	76	3	Isfahan–Iran (MT559835, MT559834, MT559833)	–	[19]
*D. n. tichomirowi*	77	3	Zanjan–Iran (MT559832, MT559830, MT559828)	–	[19]
*D. n. tichomirowi*	78	3	Eastern Azerbaijan–Iran (MT559831, MT559829, MT559827)	–	[19]
*D. n. heptneri*	79	1	Russia (KF699219)	–	[19]
*D. n. kurdistanicus*	80	1	Karmanshah–Iran (MT559871)	–	[19]
*D. n. kurdistanicus*	81	1	Kurdistan–Iran (MT559870)	–	[19]
*D. n. kurdistanicus*	82	1	Kurdistan–Iran (MT559869)	–	[19]
*D. n. kurdistanicus*	83	2	Kurdistan–Iran (MT559868, MT559860)	–	[19]
*D. n. kurdistanicus*	84	1	Kurdistan–Iran (MT559867)	–	[19]
*D. n. kurdistanicus*	85	4	Kurdistan–Iran (MT559866, MT559864, MT559861, MT559858)	–	[19]
*D. n. kurdistanicus*	86	3	Kurdistan–Iran (MT559865, MT559863, MT559862)	–	[19]
*D. n. kurdistanicus*	87	1	Kurdistan–Iran (MT559859)	–	[19]
*D. n. bilkjewiczi*	88	1	Northern Khorasan–Iran (MT559857)	–	[19]
*D. n. bilkjewiczi*	89	1	Northern Khorasan–Iran (MT559856)	–	[19]
*D. n. bilkjewiczi*	90	5	Northern Khorasan–Iran (MT559855, MT559852, MT559850, MT559847), Razavi Khorasan–Iran (MT559845)	–	[19]
*D. n. bilkjewiczi*	91	1	Northern Khorasan–Iran (MT559854)	–	[19]
*D. n. bilkjewiczi*	92	1	Northern Khorasan–Iran (MT559853)	–	[19]
*D. n. bilkjewiczi*	93	2	Northern Khorasan–Iran (MT559851, MT559848)	–	[19]
*D. n. bilkjewiczi*	94	1	Northern Khorasan–Iran (MT559849)	–	[19]
*D. n. bilkjewiczi*	95	1	Northern Khorasan–Iran (MT559846)	–	[19]
*D. n. bilkjewiczi*	96	1	Razavi Khorasan–Iran (MT559844)	–	[19]
*D. laniger* (L1)	97	1	Elmalı–Antalya (5631)	29	PV217019
*D. laniger* (L1)	98	9	Madenköy–Niğde (2321, 4579, 4586, 4587, 4588), Bolkar–Niğde (5771, 5773, 5774, 5775)	30, 31	PV217020
*D. laniger* (L1)	99	1	Bolkar–Niğde (5772)	31	PV217021
*D. laniger* (L1)	100	1	Bolkar–Niğde (5778)	31	PV217022
*D. laniger* (L1)	101	1	Bolkar–Niğde (5989)	31	PV217023
*D. laniger* (L1)	102	2	Bolkar–Niğde (6002, 6004)	31	PV217024
*D. laniger* (L2)	103	5	Kahramanmaraş (7456, 7457, 7458, 7461, 7462)	32	PV217025
*D. laniger* (L2)	104	2	Malatya (7484, 7485)	33	PV217026
*M. musculus*	105		JX457720	–	–
*M. musculus*	106		JX457718	–	–
*R. norvegicus*	107		AB746365	–	–
*R. norvegicus*	108		AB746363	–	–
*G. japonicus*	109		D89004	–	–
*G. japonicus*	110		D89001	–	–
*G. murinus*	111		AJ225115	–	–
*G. kelleni*	112		MN616984	–	–
*E. melanurus*	113		HE614012	–	–
*E. melanurus*	114		HE614010	–	–
*E. quercinus*	115		GQ453669	–	–
*E. quercinus*	116		GQ453668	–	–
*G. glis*	117		AJ225031	–	–
*G. glis*	118		KF699243	–	–
*S. anomalus*	119		AB292677	–	–
*S. anomalus*	120		AB292676	–	–
*S. vulgaris*	121		AB775131	–	–
*S. vulgaris*	122		JQ395054	–	–
*M. avellanarius*	123		MT410887	–	–
*M. avellanarius*	124		AJ225117	–	–
